# Impact of Migration and Acculturation on Prevalence of Type 2 Diabetes and Related Eye Complications in Indians Living in a Newly Urbanised Society

**DOI:** 10.1371/journal.pone.0034829

**Published:** 2012-04-10

**Authors:** Yingfeng Zheng, Ecosse L. Lamoureux, M. Kamran Ikram, Paul Mitchell, Jie Jin Wang, Christine Younan, Ainur Rahman Anuar, E-Shyong Tai, Tien Y. Wong

**Affiliations:** 1 Singapore Eye Research Institute, Singapore National Eye Centre, Singapore, Singapore; 2 State Key Laboratory of Ophthalmology, Zhongshan Ophthalmic Center, Sun Yat-sen University, Guangzhou, China; 3 Centre for Eye Research Australia, University of Melbourne, Royal Victorian Eye and Ear Hospital, Melbourne, Australia; 4 Departments of Epidemiology and Ophthalmology, Erasmus Medical Center, Rotterdam, the Netherlands; 5 Saw Swee Hock School of Public Health, National University of Singapore, Singapore, Singapore; 6 Centre for Vision Research, University of Sydney, Sydney, Australia; 7 Faculty of Medicine, University of Malaya Eye Research Centre, University of Malaya, Kuala Lumpur, Malaysia; 8 Department of Medicine, Yong Loo Lin School of Medicine, National University of Singapore, Singapore, Singapore; 9 Department of Ophthalmology, Yong Loo Lin School of Medicine, National University of Singapore, Singapore, Singapore; German Diabetes Center, Leibniz Center for Diabetes Research at Heinrich Heine University Duesseldorf, Germany

## Abstract

**Background:**

Health of migrants is a major public health challenge faced by governments and policy makers. Asian Indians are among the fastest growing migration groups across Asia and the world, but the impact of migration and acculturation on diabetes and diabetes-related eye complications among Indians living in urban Asia remains unclear.

**Methodologies/Principal Findings:**

We evaluated the influence of migration and acculturation (i.e., migration status and length of residence) on the prevalence of type-2 diabetes mellitus (T2DM) and diabetes-related eye complications (diabetic retinopathy (DR) and cataract), among first-generation (defined as participant born in India with both parents born in India, n = 781) and second-generation (participants born in Singapore with both parents born in India, n = 1,112) Indian immigrants from a population-based study of Adult Indians in Singapore. Diabetes was defined as HbA1c≥6.5%, use of diabetic medication or a physician diagnosis of diabetes. Retinal and lens photographs were graded for the presence of DR and cataract. Compared to first generation immigrants, second generation immigrants had a higher age- and gender-standardized prevalence of T2DM (34.4% versus 29.0%, p<0.001), and, in those with T2DM, higher age- and gender-standardized prevalence of DR (31.7% versus 24.8%, p<0.001), nuclear cataract (13.6% versus 11.6%, p<0.001), and posterior sub-capsular cataract (6.4% versus 4.6%, p<0.001). Among first generation migrants, longer length of residence was associated with significantly younger age of diagnosis of diabetes and greater likelihood of having T2DM and diabetes-related eye complications.

**Conclusion:**

Second generation immigrant Indians and longer length of residence are associated with higher prevalence of diabetes and diabetes-related complications (i.e., DR and cataract) among migrant Indians living in Singapore. These data highlight potential worldwide impacts of migration patterns on the risk and burden of diabetes.

## Introduction

Type 2 diabetes mellitus (T2DM) is recognised as a major chronic disease affected by lifestyle and behavioural risk factors such as obesity and physical inactivity [1]. There has been a long-standing hypothesis that rising prevalence of T2DM in many countries is related to immigration patterns from developing countries and lifestyle changes from “traditional” to “Western” patterns. People moving from developing countries to an industrialized western society are more susceptible to not only an unhealthy lifestyle (e.g., increase in fast food, smoking, and lack of exercise), but also stress factors such as air pollution, crowded living condition, and psychosocial forces. These factors may have direct or distal impacts on the development of T2DM [1], [2], [3]. However, the exact impact of migration and acculturation (the process of adaptation and exchange of behaviour patterns to the principal culture in the new country) on diabetes is not fully understood [2]. For example, a greater level of acculturation is associated with a higher prevalence of diabetes among non-Mexican Hispanics and Japanese Americans [4], [5], but a greater level of acculturation is associated with a lower diabetes prevalence in Arab Americans [6].

By absolute numbers, India is the country with the world's largest number of people affected by diabetes [7], [8]. Indians living in India have been reported to be at high risk of T2DM [7], [8], but there is a paucity of data among first- and second-generation migrant Indians, particularly those living in urban East Asian countries, where an epidemic of diabetes is also emerging [9]. Furthermore, only limited information is available on the impact of migration and acculturation status on the risk of diabetic retinopathy (DR) and cataract, the two most frequent diabetes-related eye complications and also major causes of visual impairment [10], [11].

The aim of this report is two-fold. First, we compared the prevalence of T2DM and, among those with diabetes, DR and cataract between first- and second-generation migrant Indians living in Singapore, a major migration destination for Asians. Second, we analysed the effects of acculturation, quantified by the length of residence in Singapore, on the prevalence of T2DM, DR and cataract (among persons with diabetes) in Indian immigrants. These data have potentially important implications in understanding the impact of migration, societal and related heath disparities attributable to diabetes in migrant populations in many developed countries around the world, including the US and UK.

## Methods

### Ethnics Statement

The study adhered to the Declaration of Helsinki; ethics approval was obtained from the Singapore Eye Research Institute Institutional Review Board. All participants gave written informed consent.

### Study Design and Procedure

The Singapore Indian Eye Study (SINDI) is a population-based, cross-sectional study of 3,400 Indian adults aged 40 years or older living in Singapore [12], [13]. The term “Singaporean Indians” refers broadly to people from the Indian subcontinent, including India, Pakistan, Bangladesh, and Sri Lankan (e.g., Tamils, Malays, Punjabis, Bengalis, Singhalese) who migrated to Singapore, mostly during the 19th century. Detailed population selection and methodology have been published elsewhere [13]. Briefly, between 2007 and 2009, the study was conducted in the south-western part of Singapore, using the same study protocol and the same sampling areas as the Singapore Malay Eye Study [14], [15]. An age-stratified random sampling strategy was used and 6,350 names were selected. Of these, 4,497 individuals were deemed eligible to participate and 3,400 participants took part in the study, giving a 75.6% participation rate. Of the nonparticipants, 1,021 (22.7%) declined participation and 76 (1.7%) were not contactable. Nonparticipants on average were slightly older than participants (P<0.001), but there were no gender differences (P<0.28) [13].

Participants were defined as “first-generation Indian immigrants” if they were born in India and their parents were both born in India. Participants were defined as “second-generation Indian immigrants” if they were born in Singapore and their parents were both born in India. These two samples were analysed in the current study.

### Type 2 Diabetes Mellitus and Diabetic Retinopathy Assessments

Diabetes was defined as self-report of a previous diagnosis of the disease by a doctor, use of diabetic medication, or haemoglobin A1c (HbA1c) of 6.5% or greater (as recommended by the American Diabetes Association) [1]. Participants were considered to have type-1 diabetes if they were younger than 30 years when diagnosed with diabetes and were receiving insulin therapy. Otherwise, participants were considered to have T2DM. Pre-diabetes was diagnosed in participants without diabetes but with HbA1c levels from 5.7 to 6.4% [1].

Retinal photography was undertaken using a standardised protocol [16]. After pupil dilation, one retinal photograph centred at the optic disc and another centred on the macula were taken from both eyes using a digital retinal camera (Canon CR-DGi with a 10-D SLR back; Canon, Tokyo, Japan). Photographs were sent to the University of Sydney and retinopathy lesions were graded according to a scale modified from the Airlie House classification system [16]. Retinopathy severity was categorised into minimal non-proliferative diabetic retinopathy (NPDR; level 15 through 20), mild NPDR (level 35), moderate NPDR (level 43 through 47), severe NPDR (level 53), and proliferative diabetic retinopathy (PDR, level≥60). Severity scores for the worse of the 2 eyes were used for each individual. If the images in one eye were ungradable, the scores for the fellow eye were used to define these outcomes.

### Definition of Cataract and Cataract Surgery

Slit-lamp photographs (Topcon SL-7e camera; Topcon Optical Co, Tokyo, Japan) were taken to grade the presence of nuclear cataract (NC). Retro-illumination photographs were taken (Neitz CT-R camera; Neitz Instruments Co, Tokyo, Japan) to determine the presence of cortical cataract (CC) and posterior sub-capsular cataract (PSC). According to the Wisconsin Cataract Grading System [17], [18], presence of NC was defined as opacity greater than standard. CC and PSC were determined by estimating the proportion of lens areas (by laying a grid over the anterior and posterior photographs); CC was defined if 5% or more of the total lens area was involved and PSC if any such opacity was present. Presence of cataract surgery was defined as absence of crystalline lens in at least one eye.

### Measurement and Definitions of Risk Factors

All participants underwent a detailed interview; information on birthplace, length of residence in Singapore, socioeconomic position (i.e., education, income, and housing type) [19], lifestyle risk factors (e.g., smoking), medication use and self-reported history of systemic disease was collected. Participants were asked if a health provider had ever told them that they have diabetes. Those who responded “yes” were classified as having “known diabetes”. Body mass index (BMI) was defined as weight divided by the square of height in meters (kg/m^2^). Obesity was defined as BMI greater than 25 kg/m^2^ (Indian adult population standard) [20]. Systolic and diastolic blood pressures were measured using a digital automatic blood pressure monitor (Dinamap model Pro100V2; Criticon GmbH, Norderstedt, Germany), following the protocol used in the Multi-Ethnic Study of Atherosclerosis [16]. Hypertension was defined as systolic blood pressure (SBP) of 140 mmHg or more or a diastolic blood pressure (DBP) of 90 mmHg or more, or the use of antihypertensive medication. Non-fasting venous blood samples were drawn and sent for biochemistry tests, including analysis of total cholesterol, high density lipoprotein cholesterol (HDL), low density lipoprotein cholesterol (LDL), triglycerides, glucose, and HbA1c. HbA1c was measured by high-performance liquid chromatography (HPLC).

### Statistical Analysis

Statistical analyses were performed using STATA version 11.0 (Stata Corp, College Station, Tex., USA) and R (version 2.12.1; http://cran.r-project.org). Age- and gender-standardized prevalence estimates were calculated using the 2010 Singapore population census. Binary logistic regression models were used to examine the associations of acculturation factors (including migration status and length of residence) with T2DM and DR. For multivariate analysis, only age, gender, and factors that were significantly different in univariate comparison (P<0.05) were retained in the model. Interaction effects (different combinations of the following variables: gender, generation status, and socioeconomic status) were investigated and excluded if the effects were not statistical significant. A generalized additive model (GAM, based on GAMLSS package in R) [21] with Loess smoother function was used to determine non-linear relationships of the length of Singapore residence with T2DM, DR and cataract (among those with diabetes). Possible risk factors were included as covariates in the GAM models. The Akaike Information Criterion (AIC) was used to select influential covariates in a stepwise backward fashion; any covariate would be excluded if it would result in a better model fit (the model with a lowest AIC). The continuous covariates (e.g., age, HbA1c, diabetes duration, SBP) were fitted with either a linear function or a Loess smoothing function, whereas dichotomized covariates (e.g., gender, education) were fixed factors.

## Results

There were 781 first-generation and 1,112 second-generation Asian Indian immigrants, selected from the SINDI sample, who completed the questionnaires and had their retinal photographs taken. Among first-generation immigrants, the average duration of residence in Singapore was 39.4 years (standard deviation [SD] = 18.2). Their father's birthplaces mainly included Tamil Nadu (34.9%), Kerala (6.7%), and Punjab (7.9%), and their mother's had similar birthplace distributions. Compared to first-generation immigrants, second-generation immigrants were generally younger, had higher levels of BMI, total cholesterol, HDL, LDL, and socioeconomic status, and were more likely to be female and smokers (**Table S1 and Table S2**).

**Figure 1:**
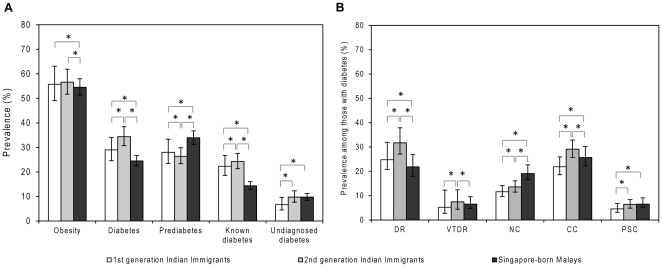
Prevalence of obesity, type-2 diabetes, diabetic retinopathy, and cataract in Indian Immigrants and local Malays living in Singapore. Asterisk indicates statistical significance between groups in age and gender adjusted regression model (p<0.05). DR = Diabetic retinopathy; VTDR = vision-threatening diabetic retinopathy; NC = nuclear cataract; CC = cortical cataract; PSC = posterior sub-capsular cataract. Prevalence data are age and gender standardized using the 2010 Singapore Indian population census.

Compared with first generation immigrants, second generation immigrants were also more likely to have higher age- and gender-standardized prevalence of T2DM (29.0% vs. 34.4%), known diabetes (22.3% vs. 24.2%), undiagnosed diabetes (6.7% vs. 9.7%), DR (24.8% vs. 31.7%), VTDR (5.2% vs. 7.5%), NC (11.6% vs. 13.6%), CC (21.9% vs. 29.1%), and PSC (4.6% vs. 6.4%). By contrast, second generation immigrants had lower prevalence of pre-diabetes (28.0% vs. 26.4%) (**Figure 1**). In multivariate logistic regression models controlling for the effects of age, gender and other major risk factors, second generation immigrants had higher prevalence of T2DM (OR = 1.29; 95%CI: 1.03, 1.62), DR (OR = 1.73; 95%CI: 1.02, 2.92), and among those with diabetes, higher prevalence of NC (OR = 1.07; 95%CI: 1.04, 1.10) and PSC (OR = 2.50; 95%CI: 1.29, 4.87) than first generation immigrants (**Table 1**), although migration status was not associated with CC (among those with diabetes: OR = 1.31, *P* = 0.22) and cataract surgery (among those with diabetes: OR = 1.08, *P* = 0.70).

**Table 1:** Associations of type-2 diabetes and diabetes-related complications with migration status.

	Age and gender-adjusted OR (95%CI)	Multivariate-adjusted OR (95%CI)
Type-2 diabetes		
1^st^ generation	Reference	Reference
2^nd^ generation	**1.24 (1.01 to 1.52)**	1.29 (1.03 to 1.62)* a
Diabetic retinopathy among diabetes patients		
1^st^ generation	Reference	Reference
2^nd^ generation	**1.29 (1.04 to 1.61)**	1.73 (1.02 to 2.92)* b
Nuclear cataract among diabetes patients		
1^st^ generation	Reference	Reference
2^nd^ generation	**1.68 (1.04 to 2.74)**	1.07 (1.02 to 2.94)* c
Cortical cataract among diabetes patients		
1^st^ generation	Reference	Reference
2^nd^ generation	1.38 (0.91 to 2.08)	1.31 (0.85 to 2.03)^c^
PSC among diabetes patients		
1^st^ generation	Reference	Reference
2^nd^ generation	**2.51 (1.34 to 4.69)**	2.50 (1.29 to 4.87)* c

OR = odds ratio; 95%CI = 95% confidence interval; PSC = posterior sub-capsular cataract. Asterisk indicates statistical significance in multivariate model (p<0.05).

a : Multivariate logistic model adjusted for age, gender, body mass index (BMI), systolic blood pressure (SBP), diastolic blood pressure (DBP), high-density lipoprotein cholesterol (HDL), low-density lipoprotein cholesterol (LDL), triglyceride, education, income, and housing type.

b : Multivariate logistic model adjusted for age, gender, BMI, SBP, DBP, duration of diabetes, hba1c level, education, income, and housing type.

c : Multivariate logistic model adjusted for age, gender, BMI, duration of diabetes, hba1c level, education, income, and housing type.

**Figure 2:**
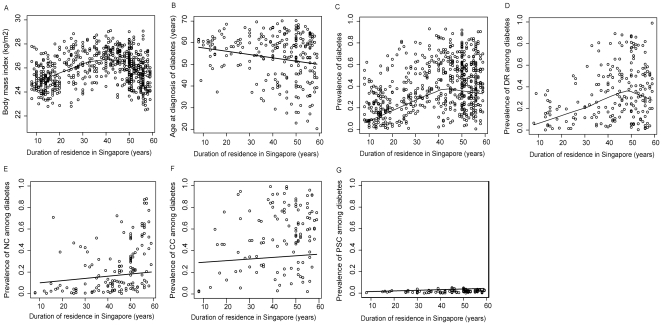
Non-linear relationships of duration of residence with prevalence of type-2 diabetes and its related complications in the first-generation Indian immigrants. Each plot is derived from a multivariate generalized additive model. The solid lines represent fitted lowess curves. Figure 2A shows the nonlinear relationship with BMI, after controlling for the influences of age, gender, systolic blood pressure (SBP), high-density lipoprotein (HDL), and low-density lipoprotein (LDL); Figure 2B shows the nonlinear relationship with prevalence of diabetes, after controlling for the influences of age, gender, BMI, SBP, HDL, LDL, triglycerides, education, income and housing type; Figure 2C shows the linear relationship with age at diagnosis of diabetes, after controlling for the influences of age, gender, BMI, SBP, hba1c level, education, income and housing type; Figure 2D shows the nonlinear relationship with prevalence of DR, after controlling the influences of age, gender, diabetic duration, hba1c level, SBP, education, income and housing type; Figures 2E to 2G show the nonlinear relationships with prevalence of nuclear cataract (NC), cortical cataract (CC), posterior sub-capsular cataract (PSC) after controlling the influences of age, gender, diabetic duration, hba1c level, education, income and housing type.

Among first-generation immigrants, longer length of residence in Singapore (as an independent continuous variable, per year increase) was significantly associated with higher prevalence of T2DM (OR, 1.03; 95%CI: 1.00, 1.06) and younger age at diagnosis of diabetes (beta coefficient  = −0.103; 95%CI: −0.207, −0.001), and among those with diabetes, higher prevalence of DR (OR, 1.04; 95%CI: 1.00, 1.10), NC (OR = 1.08; 95%CI: 1.05, 1.23)and CC (OR = 1.05; 95%CI: 1.02, 1.35) in multivariate logistic regression models. Length of residence was not significantly associated with presence of PSC and cataract surgery (*P*>0.05 for both). We also used GAM models to explore the possible non-linear relationships of length of Singapore residence (as an independent variable) with T2DM and diabetes-related ocular complications in the first generation immigrants. We showed that BMI level, prevalence of diabetes, and prevalence of DR increased in a linear fashion and then declined after more than 40–50 years of residence in Singapore. Age of diagnosis of diabetes declined in a linear manner with longer duration of residence in Singapore. Prevalence of NC and CC increased in a linear manner with longer duration of residence in Singapore (**Figure 2**).

## Discussion

In this large population-based study of immigrant ethnic Indians living in Singapore, we showed that second generation immigrants had a higher prevalence of T2DM, DR and cataract (including NC and PSC) than first generation immigrants, while controlling for BMI, socio-economic profiles and other risk factors. Consistently, amongst the first-generation immigrants, the prevalence rates of obesity, T2DM, DR, and cataract (including NC and CC) were generally higher with increasing length of residence (assimilation) in Singapore. Even the first generation immigrants had a slightly higher prevalence of T2DM than Indians living in urban southern India (29.0% versus 25.7%, standardized to the Singapore Indian population) [22]. The excess risk in the first generation may reflect a selection effect on immigration, or more likely an effect of assimilation after they moved to Singapore, a change from a “traditional” to a “western” environment [22].

The origins of this acculturation effect are multi-factorial and difficult to identify, but they may largely stem from lifestyle changes. We found that the second generation was more likely to have higher BMI level, higher lipid levels, and to be smokers. These findings support the hypothesis that the second generation immigrants may be exposed to a more harmful environment in the Western lifestyle in Singapore (**Table S1**). Previous studies have also shown that Indian immigrants living in Western countries consume more meat products and soft drinks, and have lower levels of physical activity compared with those living in India [23]. The “unhealthy” Indian diet (rich in carbohydrates and ω-6 Polyunsaturated Fatty Acids [PUFAs]; low in fibre and ω-3 PUFAs) [24] and the tradition of serving sweets may compound the adverse health effects of sedentary lifestyle and dietary changes (e.g., increased intake of high calories, fat, and processed foods) after migrating to Singapore and other developed countries [25]. These effects may explain why one-third of adult Asian Indians in Singapore had T2DM, one of the highest reported prevalence rates from general adult populations, a level higher than those of Indians living in India and elsewhere.^22^ They may also explain why the Indian immigrants (including the first generation) had a higher prevalence of DR than do Indians living in India (DR prevalence <18%) [26], [27], [28], [29].

Interestingly, the assimilation curves for T2DM and DR did not follow a linear fashion: the prevalence rates began to plateau and even decline among those living in Singapore for more than 40 years (**Figures 2C and 2D**). Explanations for this decline may be two fold. The first is that the decline in prevalence rate is “genuine”, due to survival bias (e.g., those with obesity, T2DM, and DR are known to have an increased risk of cardiovascular mortality) and/or temporal variations that cut across cohorts (e.g., economic cycle and adoption of new anti-diabetic treatment). The second is that the decline in prevalence was not “genuine”, as a result of migration effects such as “healthy-migrant effects” (e.g., selective immigration of people less susceptible to diabetes after Singapore's independence in 1965) and/or “salmon effect” (e.g., driven by the desire to die in one's birthplace, where Indians returned to India after becoming seriously ill). Importantly, we found that diabetes patients with a longer length of residence in Singapore were more likely to be diagnosed at a younger age (**Figure 2B**); this reflects an earlier onset of T2DM or earlier detection by a physician or more likely, a combination of the two effects.

Strengths of this study include its large and representative sample size, standard assessment of a wide range of risk factors, detailed classification of the first and the second generation immigrants, high frequency of gradable photographs, and the use of standardized protocols. Limitations of this study should also be noted. First, as a study related to migration, it should be borne in mind that first-generation Indian immigrants in our cohort may be unrepresentative of the population they left. Also, our study is based on the assumption that first-generation Indian immigrants had different lifestyles or environments in India or in the country before moving to Singapore. This assumption may not hold true, as acculturation may have taken place before the Asian Indians moved to Singapore. Second, the cross-sectional design of our study limits our ability to remove the influences of cohort effect, survival bias and/or selection bias related to immigration. Longitudinal data are needed to examine the relationships of baseline lifestyles and cultural factors (upon arrival in Singapore) with the onset of T2DM and its complications. Third, information on other diabetes risk factors, such as psychological stress, unhealthy dietary practices, physical inactivity and patterns of health service utilization, are not available in this study, and therefore the possibility of residual confounding in our regression analysis could not be excluded. Fourth, our study is limited by the use of a proxy measure of acculturation (i.e., length of residence). This measure may not fully reflect the complex acculturation processes, but it places minimal cognitive demands on participants and it can be easily translated. Fifth, among the diabetes patients, the presence of cataract may not be fully attributable to diabetes. However, this limitation does not invalidate our conclusion concerning the effect of acculturation on diabetes-related cataract. Finally, the relatively small number of people affected by DR limits our ability to examine the influences of migration and acculturation on PDR, macular oedema, VTDR, and other diabetic complications. Finally, in large-scale epidemiological settings, it is generally difficult to conduct careful plasma glucose measurements (e.g., fasting glucose concentration and oral glucose tolerance test) for every participant. We followed the American Diabetes Association (ADA) recommendation by using a HbA1c cut-off of 6.5% or greater for the definition of undiagnosed diabetes. The use of this cut-off may misclassify some patients at risk of diabetes, leading to non-differential misclassification, and thus may have biased some of the findings towards null.

In summary, we show that among migrant Asian Indians living in Singapore, second generation immigrant Indians have higher prevalence of T2DM, DR, and cataract than first generation immigrants. Our study shows that acculturation is an independent determinant of these diseases. This information is useful for formulating policy and designing health care programs (e.g., changing food and built environment) for the millions of migrant populations around the world, including the US and UK.

## Supporting Information

Table S1 **Characteristics of the first- and second-generation Indian immigrants with and without diabetic retinopathy living in Singapore.**
(DOC)

Table S2 **Characteristics of the first- and second-generation Indian immigrants with and without cataract living in Singapore.**
(DOC)
