# Supplementary material for: Impact of Migration and Acculturation on Prevalence of Type 2 Diabetes and Related Eye Complications in Indians Living in a Newly Urbanised Society
Source: PLoS One. 2012 Apr 10;7(4):e34829. doi: 10.1371/journal.pone.0034829 (PMC3323593; doi:10.1371/journal.pone.0034829)
Supplement: Table S1 — Characteristics of the first- and second-generation Indian immigrants with and without diabetic retinopathy living in Singapore. (DOC) [file pone.0034829.s001.doc]

**Table 1.** Characteristics of the first- and second-generation Indian immigrants with and without diabetic retinopathy living in Singapore.

|  | 1^st^ generation (N=781) | 2^nd^ generation (N=1112) | P value* | 1^st^ generation with T2DM (N=298) | 2^nd^ generation with T2DM (N=413) | P value* | 1^st^ generation with DR (N=92) | 2^nd^ generation with DR (N=134) | P value* |
| --- | --- | --- | --- | --- | --- | --- | --- | --- | --- |
| Age (per year) | 61.6 (11.3) | 56.4 (9.7) | <0.001 | 65.0 (9.9) | 59.2 (9.8) | <0.001 | 65.0 (9.0) | 59.5 (9.0) | <0.001 |
| Female gender | 351 (44.9) | 563 (50.7) | 0.01 | 136 (45.6) | 185 (44.8) | 0.82 | 41 (44.6) | 55 (41.0) | 0.60 |
| BMI (per kg/m^2^) | 25.8 (4.3) | 26.3 (4.8) | 0.01 | 26.5 (4.6) | 27.2 (5.0) | 0.06 | 26.6 (5.4) | 26.1 (4.4) | 0.45 |
| HbA1c (%) | 6.4 (1.2) | 6.4 (1.4) | 0.46 | 7.4 (1.3) | 7.7 (1.6) | 0.02 | 7.7 (1.5) | 8.0 (1.8) | 0.22 |
| SBP (per mmHg) | 137.2 (20.6) | 134.7 (19.2) | 0.006 | 142.5 (20.0) | 139.2 (19.7) | 0.03 | 146.0 (21.6) | 141.7 (18.7) | 0.11 |
| DBP (per mmHg) | 76.6 (9.8) | 77.1 (10.1) | 0.22 | 75.8 (9.8) | 77.0 (10.1) | 0.10 | 75.3 (9.9) | 76.5 (9.5) | 0.36 |
| Total cholesterol (per mmol/l) | 4.9 (1.0) | 5.2 (1.2) | <0.001 | 4.6 (1.0) | 4.9 (1.2) | <0.001 | 4.6 (1.0) | 4.9 (1.4) | 0.12 |
| HDL cholesterol (per mmol/l) | 1.0 (0.3) | 1.1 (0.3) | <0.001 | 1.0 (0.3) | 1.0 (0.3) | 0.48 | 1.1 (0.3) | 1.0 (0.3) | 0.52 |
| LDL cholesterol (per mmol/l) | 3.1 (0.9) | 3.4 (1.0) | <0.001 | 2.9 (0.8) | 3.1 (1.0) | <0.001 | 2.8 (0.8) | 3.1 (1.1) | 0.04 |
| Triglycerides (per mmol/l) | 1.9 (1.1) | 2.0 (1.2) | 0.21 | 2.0 (1.3) | 2.2 (1.3) | 0.11 | 2.0 (1.0) | 2.1 (1.2) | 0.52 |
| Previous myocardial infarction (yes) | 89 (11.4) | 111 (10.0) | 0.32 | 59 (19.9) | 60 (14.6) | 0.06 | 18 (19.6) | 24 (18.1) | 0.77 |
| Previous stroke (yes) | 23 (3.0) | 32 (2.9) | 0.93 | 12 (4.0) | 18 (4.4) | 0.82 | 7 (7.6) | 12 (9.0) | 0.72 |
| Current smoker (yes) | 75 (9.6) | 193 (17.4) | <0.001 | 24 (8.1) | 62 (15.0) | 0.005 | 7 (7.6) | 21 (15.7) | 0.07 |
| Age at diagnosis of diabetes (per year) | - | - | - | 56.1 (11.4) | 51.7 (10.2) | <0.001 | 50.9 (11.1) | 48.3 (8.7) | 0.05 |
| Duration of diabetes (per year) | - | - | - | 8.9 (9.1) | 7.4 (8.9) | 0.04 | 14.0 (10.2) | 10.9 (9.3) | 0.02 |
| Insulin treatment (yes) | - | - | - | 32 (13.2) | 37 (12.0) | 0.67 | 22 (25.0) | 27 (22.9) | 0.72 |
| Education (primary or less) | 413 (53.0) | 638 (57.5) | 0.048 | 183 (61.4) | 261 (63.4) | 0.60 | 63 (68.5) | 90 (67.7) | 0.90 |
| Income (<$SGD1,000) | 443 (56.7) | 560 (50.2) | 0.005 | 187 (62.8) | 238 (57.6) | 0.17 | 58 (63.0) | 86 (64.2) | 0.86 |
| Housing type (3-4 room flat or smaller) | 495 (63.5) | 691 (62.4) | 0.63 | 206 (69.1) | 261 (63.4) | 0.11 | 70 (76.1) | 89 (66.4) | 0.12 |

T2DM=type-2 diabetes; DR=diabetic retinopathy; BMI=Body mass index; SBP = systolic blood pressure; HbA1C = hemoglobin A1C; HDL = high-density lipoprotein; LDL = low-density lipoprotein; SGD=Singapore dollar.

Data presented are means (standard deviations) or number (%), as appropriate for variable.

*P value, comparing the differences between the 1st and 2nd generation immigrants, based on chi-square test or t test, as appropriate.
