# Supplementary material for: Impact of Migration and Acculturation on Prevalence of Type 2 Diabetes and Related Eye Complications in Indians Living in a Newly Urbanised Society
Source: PLoS One. 2012 Apr 10;7(4):e34829. doi: 10.1371/journal.pone.0034829 (PMC3323593; doi:10.1371/journal.pone.0034829)
Supplement: Table S2 — Characteristics of the first- and second-generation Indian immigrants with and without cataract living in Singapore. (DOC) [file pone.0034829.s002.doc]

**Table 2.** Characteristics of the first- and second-generation Indian immigrants with and without cataract living in Singapore

|  | 1^st^ generation diabetes patients with NC (N=69) | 2^nd^ generation diabetes patients with NC (N=83) | P value* | 1^st^ generation diabetes patients with CC (N=115) | 2^nd^ generation diabetes patients with CC (N=155) | P value* | 1^st^ generation diabetes patients with PSC (N=19) | 2^nd^ generation diabetes patients with PSC (N=43) | P value* |
| --- | --- | --- | --- | --- | --- | --- | --- | --- | --- |
| Age (per year) | 70.4 (6.2) | 67.0 (8.2) | 0.005 | 67.6 (7.2) | 62.3 (9.4) | <0.001 | 67.6 (7.6) | 66.2 (9.6) | 0.57 |
| Female gender | 31 (44.9) | 41 (49.4) | 0.58 | 63 (54.8) | 72 (46.5) | 0.18 | 10 (52.6) | 20 (46.5) | 0.66 |
| BMI (per kg/m^2^) | 26.0 (4.3) | 25.9 (5.9) | 0.94 | 26.3 (3.9) | 26.9 (4.6) | 0.21 | 26.3 (4.5) | 26.7 (5.0) | 0.77 |
| HbA1c (%) | 7.7 (1.4) | 7.7 (1.7) | 0.83 | 7.6 (1.4) | 7.6 (1.6) | 0.88 | 8.3 (1.7) | 7.5 (1.2) | 0.04 |
| SBP (per mmHg) | 146.7 (21.2) | 143.6 (18.6) | 0.33 | 144.2 (20.0) | 140.9 (18.7) | 0.17 | 149.1 (22.5) | 144.2 (18.5) | 0.38 |
| DBP (per mmHg) | 74.1 (8.1) | 73.5 (8.3) | 0.63 | 75.1 (9.4) | 74.8 (9.7) | 0.82 | 75.2 (8.1) | 74.3 (8.2) | 0.70 |
| Total cholesterol (per mmol/l) | 4.6 (1.0) | 4.8 (1.2) | 0.33 | 4.7 (1.1) | 4.8 (1.3) | 0.34 | 4.6 (0.8) | 4.8 (1.2) | 0.55 |
| HDL cholesterol (per mmol/l) | 1.0 (0.3) | 1.1 (0.3) | 0.07 | 1.0 (0.3) | 1.0 (0.3) | 0.39 | 0.9 (0.3) | 1.0 (0.4) | 0.14 |
| LDL cholesterol (per mmol/l) | 2.8 (0.9) | 2.9 (0.9) | 0.43 | 2.9 (0.9) | 3.0 (1.1) | 0.22 | 2.8 (0.8) | 3.0 (1.0) | 0.43 |
| Triglycerides (per mmol/l) | 2.3 (1.3) | 2.3 (1.5) | 0.97 | 2.1 (1.1) | 2.1 (1.3) | 0.90 | 2.6 (1.6) | 2.1 (1.1) | 0.14 |
| Previous myocardial infarction (yes) | 19 (27.9) | 21 (25.6) | 0.75 | 24 (21.1) | 34 (21.9) | 0.86 | 1 (5.6) | 12 (27.9) | 0.05 |
| Previous stroke (yes) | 2 (3.0) | 6 (7.2) | 0.23 | 6 (5.2) | 8 (5.2) | 0.98 | 2 (10.5) | 5 (11.6) | 0.90 |
| Current smoker (yes) | 8 (11.6) | 7 (8.4) | 0.51 | 8 (7.0) | 23 (14.8) | 0.045 | 3 (15.8) | 4 (9.3) | 0.46 |
| Age at diagnosis of diabetes (per year) | 59.2 (11.6) | 54.7 (12.2) | 0.02 | 57.6 (11.3) | 52.7 (11.2) | <0.001 | 57.4 (12.6) | 53.6 (11.6) | 0.25 |
| Duration of diabetes (per year) | 11.3 (9.6) | 12.2 (12.0) | 0.59 | 10.0 (9.3) | 9.6 (10.6) | 0.75 | 10.2 (9.4) | 12.6 (12.3) | 0.45 |
| Insulin treatment (yes) | 9 (15.3) | 5 (7.1) | 0.14 | 17 (17.5) | 13 (10.6) | 0.13 | 5 (29.4) | 5 (14.3) | 0.19 |
| Education (primary or less) | 55 (79.7) | 65 (78.3) | 0.83 | 83 (72.2) | 102 (66.2) | 0.30 | 12 (63.2) | 30 (71.4) | 0.52 |
| Income (<$SGD1,000) | 56 (81.2) | 68 (81.9) | 0.90 | 84 (73.0) | 97 (62.6) | 0.07 | 14 (73.7) | 32 (74.4) | 0.95 |
| Housing type (3-4 room flat or smaller) | 54 (78.3) | 56 (67.5) | 0.14 | 86 (74.8) | 88 (56.8) | 0.002 | 11 (57.9) | 25 (58.1) | 0.99 |

T2DM=type-2 diabetes; DR=diabetic retinopathy; BMI=Body mass index; SBP = systolic blood pressure; HbA1C = hemoglobin A1C; HDL = high-density lipoprotein; LDL = low-density lipoprotein; SGD=Singapore dollar. NC=nuclear cataract; CC=cortical cataract; PSC=posterior sub-capsular cataract.

Data presented are means (standard deviations) or number (%), as appropriate for variable.

*P value, comparing the differences between the 1st and 2nd generation immigrants, based on chi-square test or t test, as appropriate.
